# Thermal and Humidity Performance Test of Rammed-Earth Dwellings in Northwest Sichuan during Summer and Winter

**DOI:** 10.3390/ma16186283

**Published:** 2023-09-19

**Authors:** Maqi Jiang, Bin Jiang, Renzi Lu, Liang Chun, Hailun Xu, Gaolin Yi

**Affiliations:** 1 School of Civil Engineering and Architecture, Southwest University of Science and Technology, Mianyang 621010, China; 15681171997@163.com (M.J.); lrz1065@163.com (R.L.); chunliang@swust.edu.cn (L.C.); xuhailun@swust.edu.cn (H.X.); 2 Sichuan Dongsheng Engineering Design Co., Ltd., Mianyang 621010, China; yigaolin1986@163.com

**Keywords:** rammed–earth materials, thermal and humidity physical properties, heat and moisture coupling transfer, indoor environment

## Abstract

Rammed-earth dwellings have a long history in the construction field. It is a natural material that is both green and environmentally friendly. In recent years, the advantages of rammed earth, such as environmental protection, low cost, and recyclability, have attracted considerable attention. In this study, the thermal and humidity physical properties of rammed–earth materials in the northwest Sichuan region, the variation laws of thermal physical parameters, such as the thermal conductivity of rammed–earth under different moisture content conditions, and isothermal moisture absorption and desorption curves were investigated. The results indicated that the thermal physical parameters of the rammed earth measured in the experiment increased with an increase in moisture content, and its moisture absorption performance was better than the moisture release performance in the range of 11.31–97.3% relative humidity. The experimental site, Mianyang City, Sichuan Province, is a subtropical monsoon humid climate zone characterized by warm winters and hot summers with four distinct seasons. In this study, we investigated the hygrothermal coupling transfer of walls, as well as the indoor temperature and humidity changes in new rammed–earth buildings during summer and winter climates. During the test period, the maximum indoor temperature in summer was 35.08 °C, the minimum temperature was 33.76 °C, and the average daily temperature fluctuation was 3.62 °C. In winter, the maximum indoor temperature was 8.59 °C, the minimum temperature was 6.18 °C, and the average daily temperature fluctuation was 1.21 °C. An analysis was performed on the thermal insulation performance of rammed–earth buildings in an extremely high-temperature climate during summer, thermal insulation performance, the thermal–buffering capacity of walls in a low–temperature and high-humidity climate during winter, and thermal and humidity regulation of indoor environments provided by walls during summer and winter. The results showed that the rammed–earth buildings exhibited warmth in winter, coolness in summer, and a more stable and comfortable indoor environment.

## 1. Introduction

Against the backdrop of global economic integration, a rapidly developing world economy has brought about significant energy consumption, with the construction sector accounting for a large proportion [1]. This has given rise to corresponding problems, such as significant energy consumption and increased greenhouse gas emissions. The regulation of indoor thermal and humid environments accounts for the vast majority of building energy consumption, such as air conditioning, cooling, and indoor ventilation in summer [2]. In China, although energy consumption in the architectural field is less than half that of developed countries, the consumption of hot and cold energy is three times that of developed countries [3]. For sustainable development and ecological protection, China is focused on energy consumption in the architectural field, as well as the development of green building work and the improvement of indoor heat and humidity environments, which have become key considerations in the field of energy conservation [4]. In addition, most of China’s population and building area distributions are located in rural areas, and the building energy consumption in these areas accounts for 25% of the country’s total [5]. To effectively reduce energy consumption in the architectural field, it is essential to improve the thermal and hygroscopic performances of buildings [6].

Rammed earth is a very old building material, which is very common and often promoted as a sustainable building material [7]. As a local renewable material, rammed–earth does not require industrial processing and can be produced and reused at or near construction sites with very low energy consumption [8]. On the one hand, rammed–earth construction is more common and accounts for a high percentage of dwelling types in rural dwellings in southwest China [9], while the scarcity of resources, energy problems, and environmental pollution have brought people’s attention back to rammed–earth.

It is well known that rammed–earth walls can regulate indoor temperature and humidity [10], reduce energy consumption, and create low–carbon and comfortable indoor spaces. Rammed–earth walls are increasingly valued because of their low implied energy, moisture–buffering capacity, and thermal stability [11]. They have demonstrated many technical, economic, social, and environmental benefits that meet the needs for alternative building materials [12]. According to statistics, hundreds of millions of people worldwide live in houses made of rammed–earth, of which at least 60 million live in rammed–earth buildings nationwide, mostly in the western region [13].

A wide range of domestic and foreign scholars have conducted studies on the different properties of rammed earth. Fernando et al. [14] investigated the use of environmentally friendly stabilizers to enhance the compressive resistance and heat insulation capacity of rammed–earth materials. Losini et al. studied the effects of natural additives on various rammed–earth houses (gypsum, compressed clods, and rammed earth) and suggested that the use of waste materials as additives is very valuable [15]. Giuffrida Giada et al. studied evaluation methods for rammed earth regarding its thermal and humidity properties, and showed that its thermal properties depend on the moisture content; however, they did not provide a quantitative relationship [16]. There is a lack of standardized experimental protocols for evaluating the hygrothermal properties of rammed earth, which indicates that properties evaluated under different boundary conditions cannot be compared immediately. In addition, the results can be distorted by changes in the rammed earth, which is a “heterogeneous” material (soils from different areas or layers in the same quarry may have different properties), and the specimen may not reflect the real situation. Desogus et al. [17] conducted summer environmental monitoring of earthen buildings in Sardinia and studied the differences in the thermal properties between floors using an adaptive comfort model. The study showed that rammed–earth buildings can guarantee specific comfort performance in high-temperature weather without the use of air conditioning, but only for the lower floors, and the thermal properties of the upper floors of buildings can be improved by combining appropriate technical improvements such as roof insulation or window replacement techniques. However, rammed–earth dwellings are located in a Mediterranean climate region, and the results of this study are limited to summer climatic conditions; there are no other studies on rammed earth under these climatic conditions. Fernandes et al. discussed the advantages of using rammed–earth materials at each usage stage of a building and the possibility of recycling these materials in a closed–loop manner [18]. Research has shown that, compared to materials produced in industry, rammed–earth materials have more environmentally friendly advantages, lower carbon emissions, and consume less energy.

This study considered rammed–earth buildings in northwest Sichuan as a case study. First, the heat and humidity physical property parameters of rammed-earth materials were measured under different relative humidity environments, and their change laws were analyzed. Second, the heat and moisture coupling transfer of rammed–earth buildings in the winter and summer seasons was comprehensively monitored and analyzed through field tests, theoretical analysis, and other methods to study the heat and moisture performance of rammed–earth walls under typical climatic conditions and the temperature and humidity changes in building indoor environments, as well as their influence on living comfort.

## 2. Study of Thermal and Humidity Physical Properties of Rammed–Earth Materials

### 2.1. Measurement of Thermal and Humidity Physical Property Parameters of Rammed–Earth Materials

#### 2.1.1. Preparation of Test Specimens

**Figure 1:**
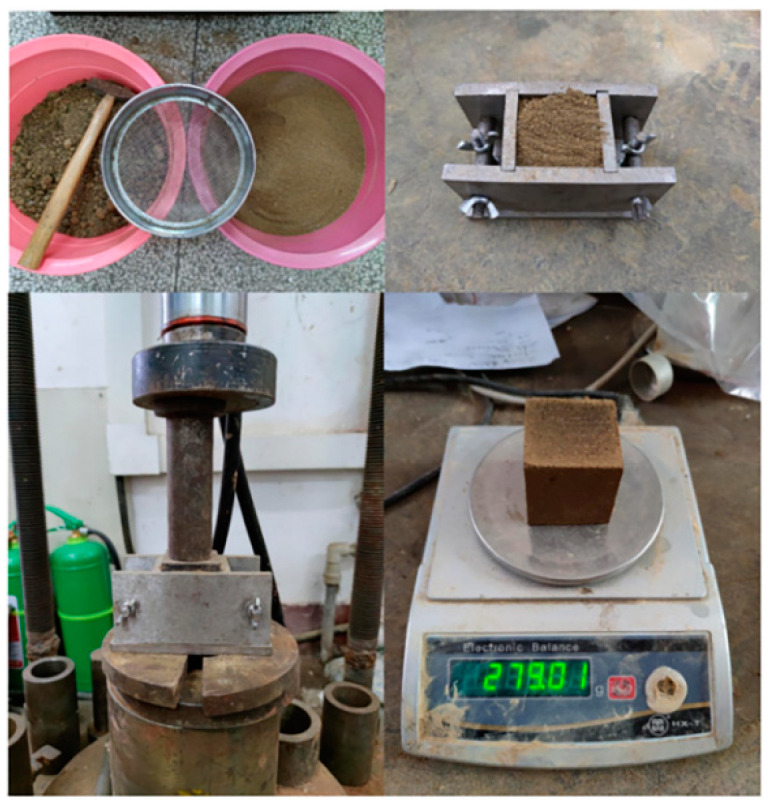
Preparation of rammed–earth specimens.

The rammed earth used for the experiment was obtained from Mianyang (104.56 E, 31.53 W), Sichuan Province, consistent with the rammed–earth used in the building, and approximately 5 kg of the sample soil was taken, crushed, sieved with a test sieve (square hole diameter of 1.6 mm), and pressed into a shape in a mold, as shown in Figure 1 [19]. A total of 18 square specimens (L = 50 mm) with a mass of approximately 280 g and a density of approximately 2240 kg/m^3^ met the strength requirements of the standard compressive strength of 2.0 MPa [20] for rammed–earth materials [21].

**Figure 2:**
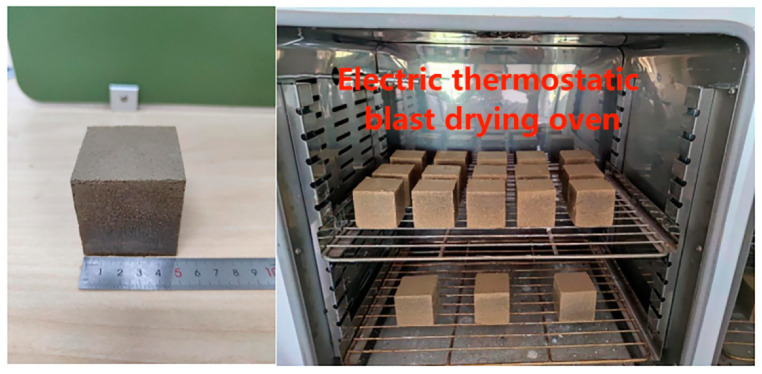
Specimen drying.

After the prepared specimens were maintained at 25 ± 0.5 °C and 60 ± 2% RH for 7 d, the specimen was dried in a dry box at 105 °C for 24 h as shown in Figure 2 (specimens intact after drying) [22,23,24], and then weighed. When weighed three times in a row and the change in specimen weight was less than 0.1%, the specimens were considered completely dry. The mass of the dried specimen was approximately 252 g and its density was approximately 2016 kg/m^3^.

#### 2.1.2. Thermal Conductivity and Thermal–Diffusion Coefficient

**Figure 3:**
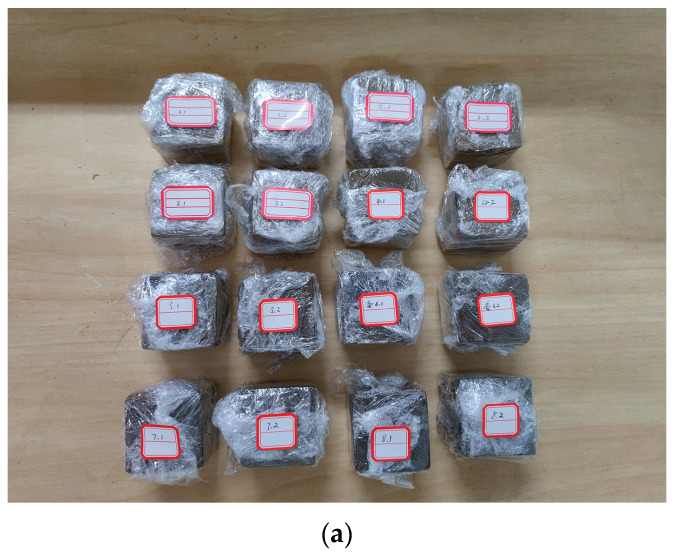

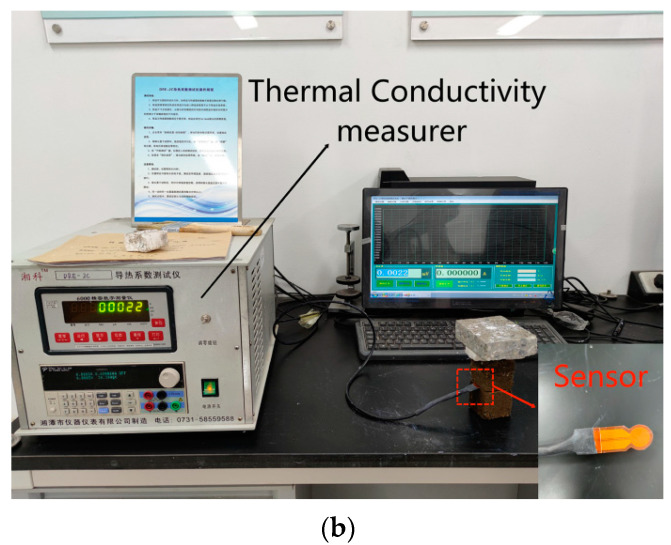
Specimen handling (**a**) and testing (**b**).

The specimens were placed on a cling film, and six faces of the specimens were continuously and randomly sprayed with pure water using a capillary nozzle. Nine groups of rammed–earth specimens with moisture content ranging from 0% to 16% (moisture content gradient of 2%) were prepared separately [25]. As shown in Figure 3, after spraying the specimen with water, it was immediately wrapped with plastic wrap. The specimen was weighed after standing for 24 h; if the quality of the specimen did not meet the experimental requirements, then the above steps were repeated until the specimen had sufficiently absorbed water to reach the target moisture content. The test method was the transient planar heat source method, with a DRE-2C thermal conductivity tester (measurement range 0.01–100 W/(m·K), accuracy: ≤±5%), under an experimental environment of 20 ± 2 °C. Each group of specimens was tested thrice, and the results were averaged.

#### 2.1.3. Isothermal Moisture Absorption and Discharge Curve

**Figure 4:**
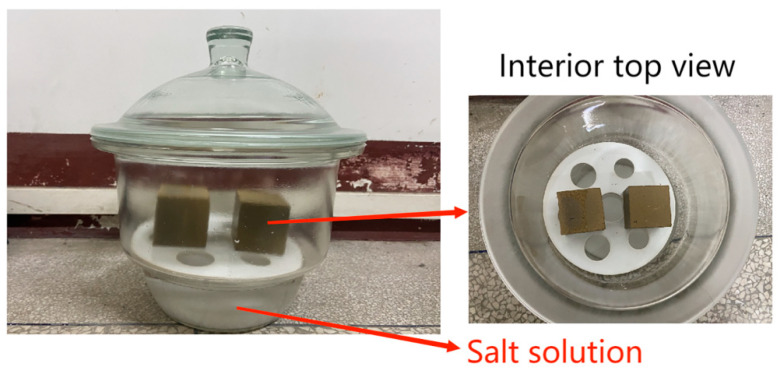
Diagram of isothermal moisture absorption and discharge curve tests.

Isothermal moisture absorption and discharge curves were plotted based on the equilibrium moisture content obtained in isothermal environments with different relative humidities. The moisture absorption and discharge capacities of the rammed–earth materials were obtained by studying their isothermal moisture absorption and discharge curves. The experiment was conducted using the desiccator method, and the indoor experimental environment was maintained at 25 ± 1 °C and 60 ± 1% relative humidity [22,23,24]. The specific experimental steps were as follows: (1) The square rammed-earth specimen was placed into the blast–drying oven at a temperature of 105 °C for 24 h, continuously (specimens intact after drying), and when the change in specimen quality was not more than 0.1%, the specimen was considered completely dry. (2) After the specimens were cooled, they were placed sequentially on partitions in drying dishes containing saturated salt solutions with low to high relative humidities for the experiments, as shown in Figure 4. The corresponding relative values of the saturated salt solutions are listed in Table 1. (3) Every 24 h, the test piece was weighed, and two consecutive weights were measured. If the mass difference was less than 0.1%, then the test piece was considered to have reached equilibrium moisture absorption. (4) The rammed–earth specimens that reached moisture absorption equilibrium were then placed in drying dishes with high to low relative humidities to conduct the moisture release process. (5) The equilibrium moisture content was calculated as follows:

*u(φ*) = (*m_i_*(*φ*) *− m*_0_)/*m*_0_, (1)

where *u*(*φ*) is the equilibrium moisture content of the specimen corresponding to the relative humidity, kg/kg; *m_i_*(*φ*) is the mass of the specimen when the relative humidity is in equilibrium, kg; *m*_0_ is the mass of the specimen when it is completely dry, kg.

### 2.2. Analysis of Thermal and Humidity Properties of Rammed-Earth Materials

#### 2.2.1. Thermal Performance Analysis

**Figure 5:**
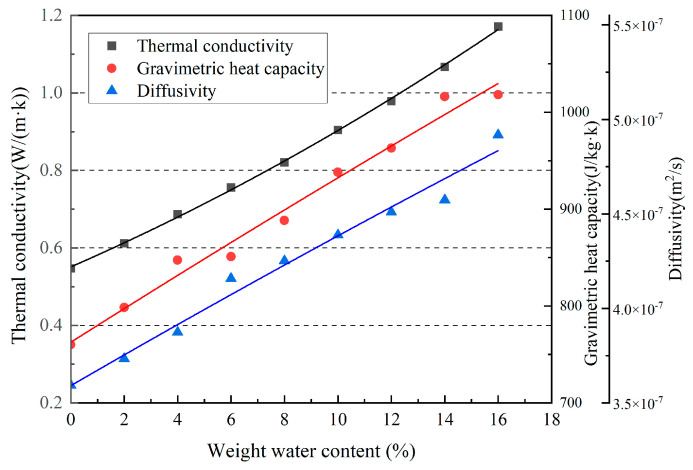
Thermal conductivity, thermal−diffusion coefficient, and mass−specific heat capacity.

The test results for the thermal conductivity and thermal–diffusion coefficient, and the calculation results for the specific heat capacity [26] are shown in Figure 5. To improve the accuracy of the fitting results, a quadratic polynomial was used to fit the experimental data, and the functional relationship between the thermal conductivity and thermal–diffusion coefficient of the rammed–earth material and water content was obtained as follows:

*λ* = 0.03*w* + 5.139*w*^2^ + 0.551, (2)

*D* = 8.18 × 10^−9^*w* − 2.57 × 10^−11^*w*^2^ + 3.591 × 10^−7^, (3)

where *λ* is the thermal conductivity (W/m·K), *D* is the thermal–diffusion coefficient (m^2^/s), and *w* is the moisture content weight.

For the same substance, the material structure, density, temperature, humidity, and pressure affect the thermal conductivity [27]. In general, solids have the highest thermal conductivities among solids, liquids, and gases, followed by liquids and gases. This is because the molecular spacings between the different states are different; thus, their thermal conductivities vary. The lower the water content and temperature of the same substance, the lower its thermal conductivity.

From Figure 5, as the moisture content of the material increases, its thermal conductivity also becomes larger. The reason is that rammed–earth materials are porous media; for non–dry porous media, the material contains more moisture than dry materials, and this moisture replaces the original air in it. At the same time, the thermal conductivity of liquid water (approximately 0.59 W/m·K) in the normal state is much greater than air (approximately 0.026 W/m·K) [28,29]. Therefore, for rammed–earth material, its thermal conductivity is positively correlated with moisture content.

The ability of a rammed–earth material to maintain its initial temperature when disturbed is called the thermal mass, and the thermal mass of materials is quantified by the thermal–diffusion coefficient. As the thermal–diffusion coefficient increases, the thermal inertia of the material decreases, enabling the surface material to reach thermal equilibrium more quickly. The formula used is as follows:

*D* = *λ*/(*ρ_m_C_p,m_*), (4)

where *D* is the thermal–diffusion coefficient (m^2^/s), *λ* is the thermal conductivity (W/m·K), *C_p_*_,*m*_ is the mass–specific heat capacity (J/kg·K), and *ρ_m_* is the density, kg/m^3^.

Based on Equation (4), using the measured thermal conductivity and thermal diffusion coefficients, the mass specific heat capacity was calculated. A quadratic polynomial was fitted to the results, and the functional relationship was obtained as follows:

*C_p_*_,*m*_ = 17.44*w* − 0.05*w*^2^ + 762.636. (5)

The experimental results showed that the thermal conductivity, thermal diffusion coefficient, and mass specific heat capacity of rammed–earth materials increased with the increase in moisture content. The thermal conductivity of the material was most affected by the moisture content, compared to the moisture content at 0% (the specimens were dried with very low moisture content, tending to 0%). The thermal conductivity improved by 114.1% when the moisture content reached 16%, and the thermal–diffusion coefficient was least affected, with an improvement of 36.9%. The greatest increase in thermal conductivity and mass specific heat capacity was observed at 4% moisture content of the rammed–earth material, with a 12.3% increase in thermal conductivity and an 8.2% increase in mass specific heat capacity, and the smallest increase in mass specific heat capacity was observed at 16% moisture content, with a 2.1% increase.

#### 2.2.2. Material Humidity Performance Analysis

There are many different analytical expressions for isothermal hygroscopic curves, and the Peleg model was used to calculate these curves [30]. The model proposed by Peleg et al. is as follows:

*U* = *aφ^b^* + *cφ^d^*, (6)

where *φ* is the relative humidity, taking a value between 0 and 1, and *U* is the equilibrium moisture content of the material, kg/kg.

**Figure 6:**
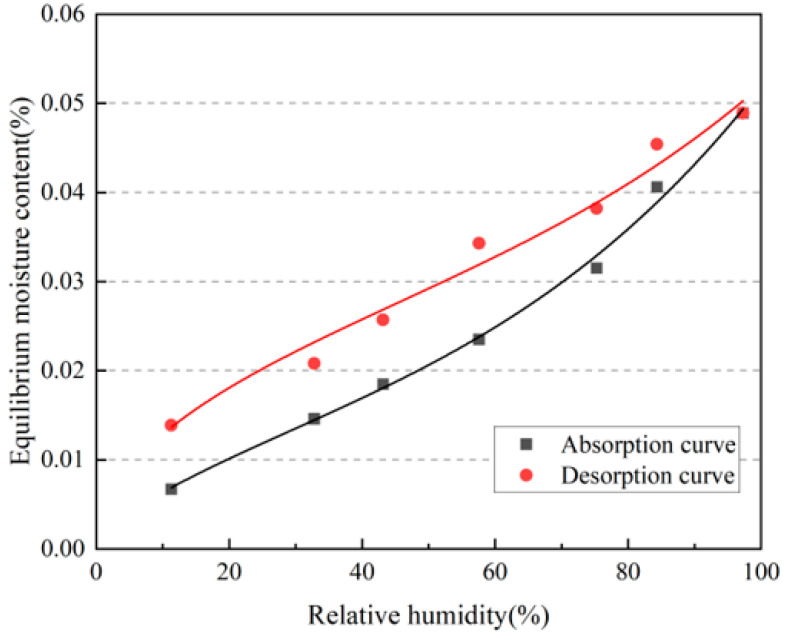
Isothermal moisture absorption and discharge curves.

As shown in Figure 6, the isothermal moisture absorption and discharge curves were obtained as functions of the relative humidity by fitting a nonlinear curve according to Equation (6).

Absorption curve: *y* = 0.0137*φ*^0.66^ + 4.31 × 10^−9^*φ*^3.36^ (7)

Desorption curve: *y* = 0.0416*φ*^0.49^ + 5.12 × 10^−10^*φ*^3.69^ (8)

## 3. Building Wall Heat and Moisture Coupling Transfer and Indoor Heat and Humidity Environment Testing

### 3.1. Test Overview and Experimental Purpose

**Figure 7:**
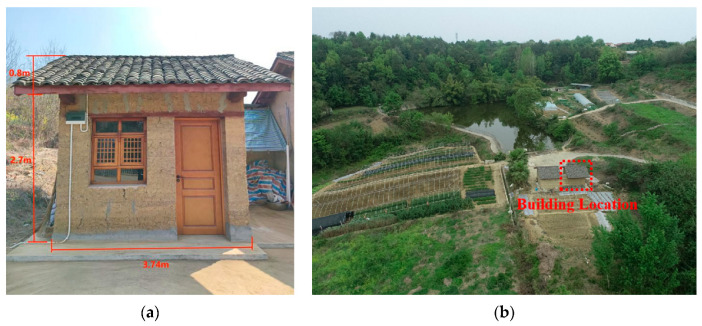
Appearance of rammed–earth building (**a**) and building surroundings (**b**).

To study the coupled heat and humidity transfer process and the indoor heat and humidity environment of the rammed–earth building envelope, the temperature and humidity were measured. The experiment was a year–round uninterrupted test. The test data from 11 August to 17 August and 1 December to 7 December 2022 were selected for the analysis and research in this paper, with the summer period having a local extreme high–temperature climate of 1 in 60 years. During the experiment, the doors and windows were closed, and there was no ventilation, indoor heat source, or moisture source. The experimental building is located at Southwest University of Science and Technology, Mianyang (104.56 E, 31.53 W), Sichuan Province. It was constructed using the slab–on–wall construction method, completed in August 2021, and is only used for experiments without human habitation. From the interior floor to the highest point of the sloped roof is 3.5 m, and to the eaves is 2.7 m. The room width is 3 m, depth is 4 m, and wall thickness is 0.37 m, as shown in Figure 7a.

### 3.2. Test Content and Equipment Layout

**Figure 8:**
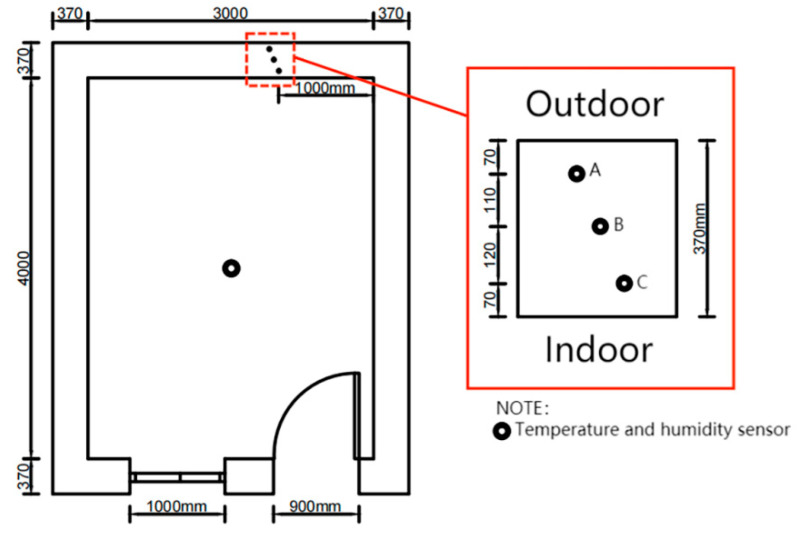
Wall internal probe arrangement.

**Figure 9:**
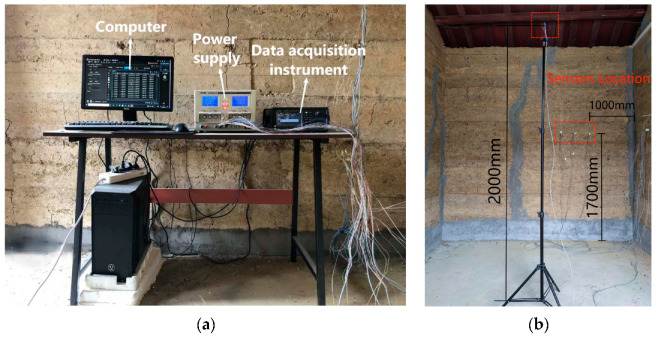
Indoor equipment (**a**) and probe location (**b**).

The test system provided real–time measurements of the indoor temperature and humidity in the buildings, internal temperature, humidity in walls, outdoor temperature and humidity, and solar radiation on the north side of the building. The experiments were conducted by collecting, recording, and storing temperature and humidity data using a Keysight-DAQ970A data acquisition instrument at a recording interval of 10 min. Temperature data were measured using a K-type thermocouple (temperature range: 0–1300 °C, accuracy: ±0.5 °C), humidity data were measured by a Honeywell capacitive humidity sensor (HIH-4000-003, accuracy: ±3.5%), and the humidity sensor was connected to a 5 V DC power supply. Solar radiation was measured by a JTR05 type solar radiation sensor (spectral range: 0.3–3.2 μm, accuracy: ≤±2%). The internal wall temperature and humidity sensors were arranged in a perforated manner at 1.7 m high and 1 m from the corner of the north wall, with the arrangement direction perpendicular to the wall, and the arrangement depths were 0.07 m(C), 0.19 m(B), and 0.30 m(A) from the internal surface of the wall, as shown in Figure 8. The indoor temperature and humidity sensors were placed at the center at a height of 2 m, as shown in Figure 9. The outdoor temperature and humidity sensors were arranged in a louvered box that was approximately 2 m away from the north side of the exterior wall and approximately 1 m high. The solar radiation meter was placed at an unobstructed location directly in front of the building.

## 4. Summer Test Results and Analysis

The test data from 00:00 on 11 August to 24:00 on 17 August in summer were selected for research, which all had typical summer high–temperature weather, and the test period consisted of continuous sunny days with no rainfall. To avoid affecting the test environment during the test, there was no heat or humidity source in the building.

### 4.1. Wall Heat and Moisture Coupling Transfer

#### 4.1.1. Wall Heat Transfer

**Figure 10:**
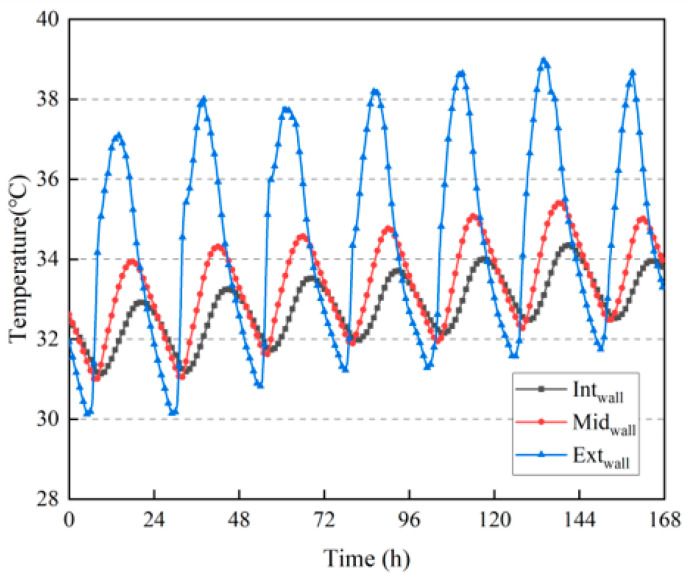
Temperature variation inside the wall.

Figure 10 shows the temperature change inside the wall, where the temperature of the outer surface of the wall fluctuated significantly. During daytime solar irradiation, the highest temperature of the outer surface was close to 40 °C, while at night, when there was no sunlight, the lowest temperature dropped to below 32 °C. It can be observed from Figure 10 that the daily temperature fluctuation of the outer surface of the wall was approximately 7.5 °C. The temperature fluctuations in the middle and inner surfaces of the walls were relatively smooth, with daily temperature fluctuations of approximately 3 °C and 1.5 °C, respectively. As listed in Table 2, the daily average temperature of the inner surface of the wall was the lowest in all cases, whereas the temperature fluctuated widely on the outer surface of the wall. The daily average temperature of the outer surface of the wall was the highest in all cases, indicating that the rammed earth had a certain heat–buffering capacity [31,32].

Because the energy performance of a building is difficult to quantify, as it depends on its inherent parameters as well as the living environment, this study investigated the energy transfer information of a building in terms of heat flux. Based on Equation (9), the heat flux of a rammed–earth wall can be calculated using test data, which was calculated as

*q_test_* = *h_i_*(*T_i_* − *T_surfi_*), (9)

where *q_test_* is the heat flux during the test period, W/m^2^; *h_i_* is the convective heat–transfer coefficient of the surface inside the wall, W/(m^2^·K); *T_i_* is the room temperature, K; *T_surfi_* is the temperature of the surface inside the wall, K.

**Figure 11:**
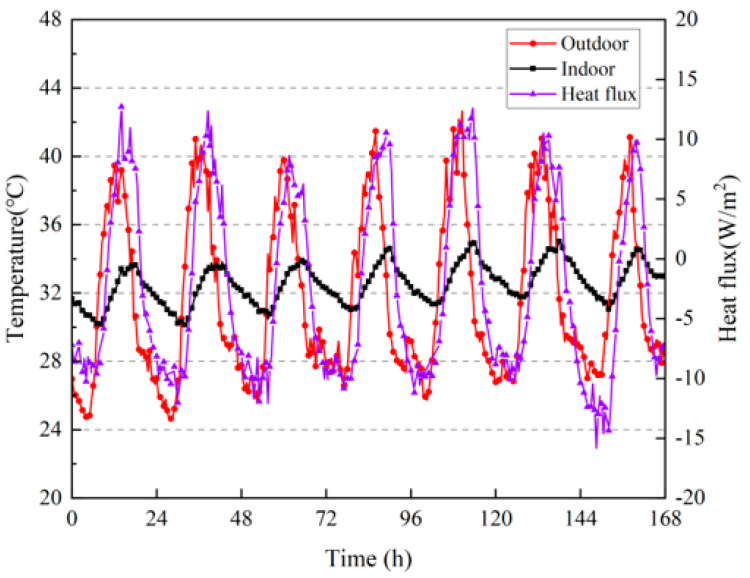
Heat fluxes on the surface inside the wall; indoor and outdoor temperatures.

Figure 11 shows the heat flux and indoor and outdoor temperatures of the internal surface of the wall. The heat flux calculation resulted in positive values, indicating that the wall absorbed heat, whereas negative values indicate that the wall exerted heat. As shown in Figure 11, during the day, when the temperature increased, the inner surface of the wall absorbed the heat from the indoor air. At night, the outdoor temperature was lower than the indoor temperature, and the wall transmitted the stored heat indoors. This spontaneous heat transfer can effectively regulate indoor temperature, reduce the need for air conditioning, and achieve the purpose of saving energy. Within 7 d of the test, the total heat absorption by the inner surface of the wall was 839.39 W/m^2^ and the total heat release was 1656.46 W/m^2^.

#### 4.1.2. Wall Moisture Transfer

**Figure 12:**
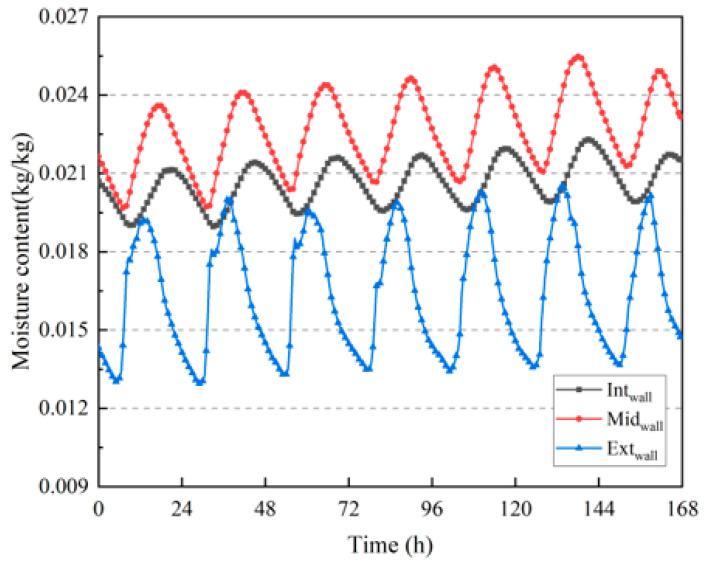
Variation in moisture content inside the wall.

The building was built in only one year, and a large amount of moisture was retained inside the wall; therefore, the moisture content was highest in the middle of the wall. According to Figure 10 and Figure 12, the larger the temperature fluctuation, the greater the moisture content fluctuation at the corresponding position inside the wall.

The moisture flux on the inner surface of the wall was calculated based on test data.

*g_test_* = *βi*(*φ_i_P_sat,I_* − *φ_surfi_P_sat,surfi_*), (10)

where *g_test_* is the wet flux during the test period (kg/m^2^), *βi* is the convective mass transfer coefficient of the surface inside the wall (s/m), *φ_i_* is the relative humidity of the room, *φ_surfi_* is the relative humidity of the internal surface of the wall (%), *P_sat_*_,*i*_ is the indoor saturated water vapor partial pressure (Pa), and *P_sat,surfi_* is the saturated water vapor partial pressure on the internal surface of the wall (Pa).

**Figure 13:**
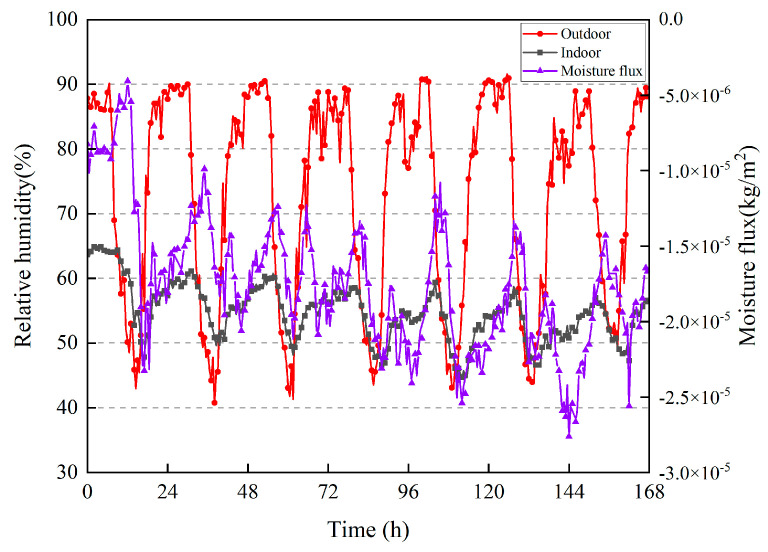
Moisture flux on the surface inside the wall; indoor and outdoor relative humidities.

Figure 13 shows the moisture flux calculation results and the indoor and outdoor relative humidities during the actual measurement period. If the calculation result is positive, then the wall absorbs moisture; otherwise, the wall dissipates moisture. Owing to the short building construction time, the moisture content of the wall was high, and the test period experienced a continuous high temperature without rainfall. During the 7 d of the test, the wall continued to release moisture into the room, and the total moisture dissipation was 0.00586 kg/m^2^.

### 4.2. Indoor Temperature and Humidity Changes

#### 4.2.1. Indoor Temperature

**Figure 14:**
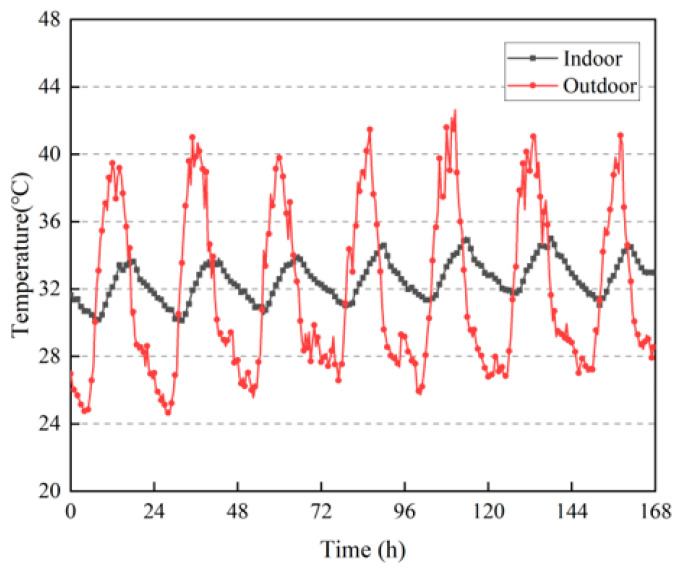
Changes in indoor and outdoor temperatures.

According to Figure 14, the outdoor temperature fluctuated significantly, with the average daily outdoor temperature fluctuating above 14 °C. The highest outdoor temperature was 42.6 °C, whereas the indoor temperature fluctuated more steadily, with the daily temperature fluctuating by no more than 4 °C, and the highest daily temperature was below 36 °C. This shows that the rammed-earth building had a good heat insulation effect and thermal stability, even under extremely high–temperature weather conditions.

Table 3 shows the highest daily indoor temperature lag time of the rammed-earth building: the average thermal lag time was 5.07 h, and the longest lag time was up to 8 h. On the fifth day of the highest outdoor temperature, there was still a thermal lag time of 3.5 h, which indicated that the rammed-earth building had good thermal–buffering capacity and thermal lag.

#### 4.2.2. Indoor Humidity

**Figure 15:**
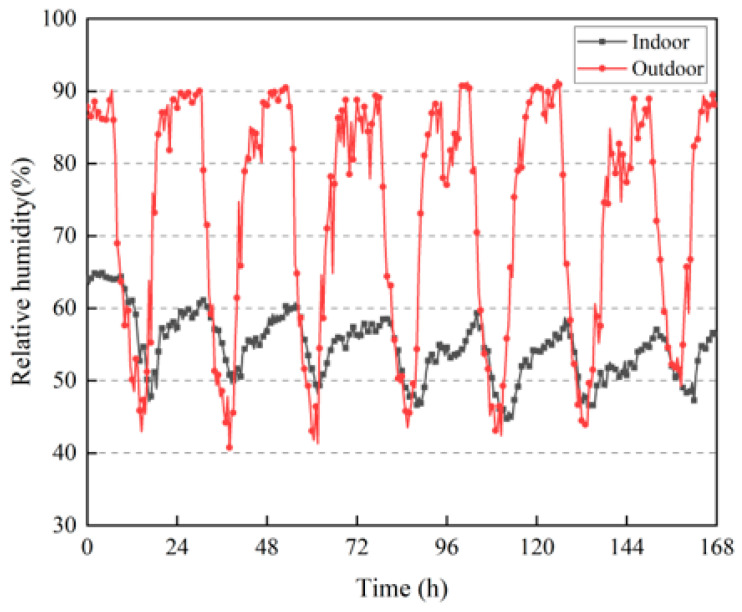
Changes in indoor and outdoor relative humidity.

**Figure 16:**
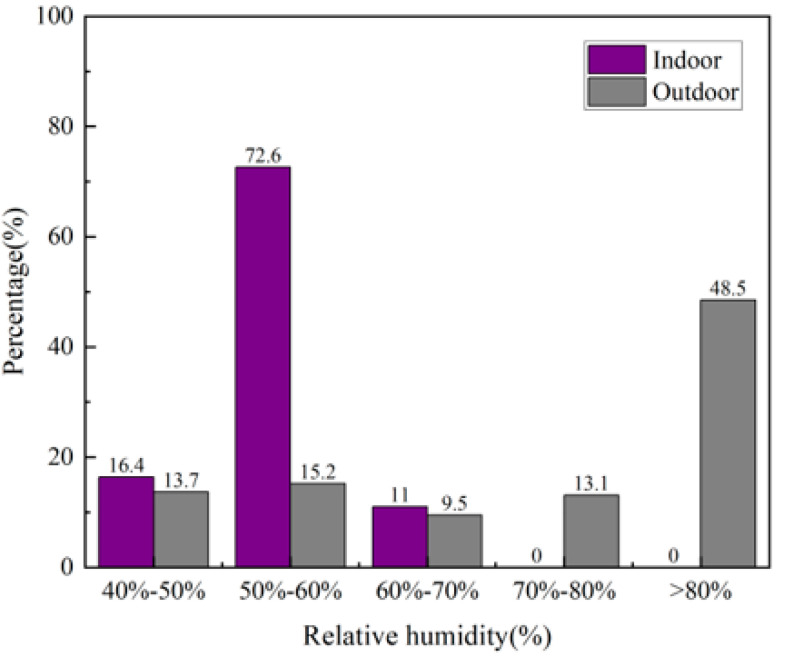
Percentage of indoor and outdoor relative humidity periods.

Indoor relative humidity is an important reference index for comfort in a living environment. As shown in Figure 15, the outdoor relative humidity fluctuated greatly, and during the daytime sunshine period, the lowest outdoor relative humidity was between 40% and 50%, owing to the high outdoor temperature and fast evaporation rate of moisture. At night, the relative humidity reached approximately 90%. This is because the building is located on the hillside, where vegetation covers a large area, and there is a large body of water near the building (there is a pond directly in front of the building), as shown in Figure 7b. When the air temperature decreases at night, the vegetation and body of water keeps dispersing moisture into the air, and the water vapor in the air at night is more likely to coalesce. Compared with the outdoor humidity, the indoor relative humidity fluctuated more smoothly, with the relative humidity fluctuating between 40% and 70%. The proportion of indoor and outdoor relative humidity periods can be obtained from Figure 16, where the indoor relative humidity is between 50% and 60% for 72.6% of the time, whereas the outdoor relative humidity is greater than 80% for 48.5% of the time. There are also proportional periods in each section between 40% and 80%, and the relative humidity periods are more dispersed. This result proves that rammed–earth buildings can regulate the relative humidity of indoor air and have a stable indoor humidity environment, which is conducive to comfortable living conditions.

## 5. Winter Test Results and Analysis

Test data from 00:00 on 1 December to 24:00 on 7 December in winter were selected for research, which all had typical winter low–temperature weather. The seventh day of the test was sunny, and the rest of the period was cloudy with no rainfall, similar to summer, with no other indoor heat or humidity sources.

### 5.1. Wall Heat and Moisture Coupling Transfer

#### 5.1.1. Wall Heat Transfer

**Figure 17:**
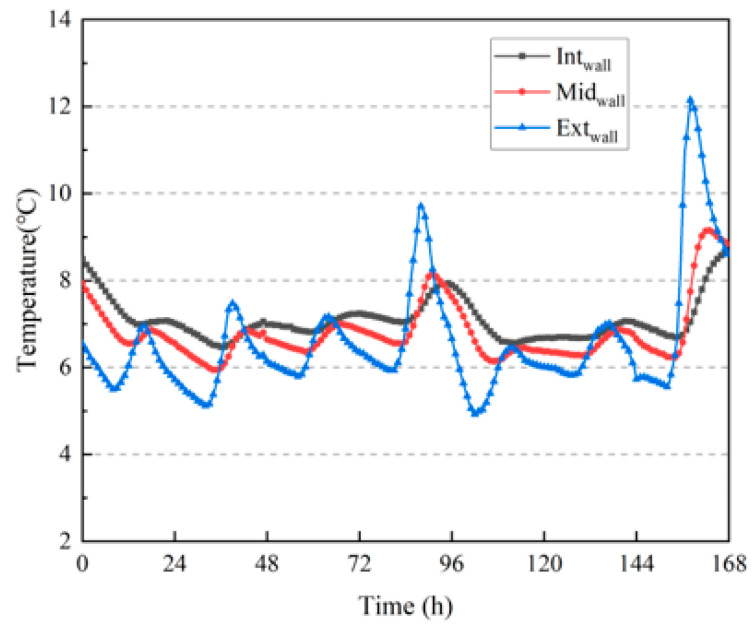
Temperature variation inside the wall.

Figure 17 shows the temperature changes inside the compacted wall during the winter. The temperature fluctuation on the outer surface of the wall was significant. However, owing to the lower temperature in winter, the fluctuation was much smaller than that in summer, and the temperature fluctuations of the wall gradually leveled off from outside to inside. Except for the seventh day, the fluctuation was approximately 1 °C. The temperature fluctuation on the outer surface of the wall was significantly greater than that on the middle and inner surfaces of the wall, with the maximum fluctuation approaching 7 °C, indicating that the rammed-earth wall also had good thermal–buffering capacity in winter.

As shown in Table 4, the daily average temperature of the inner surface of the wall was the highest six days before the test, and the inner surface temperature was slightly lower than the outer and inner surface temperatures of the wall on the seventh day. It can be concluded that the rammed-earth wall exhibited good insulation performance.

**Figure 18:**
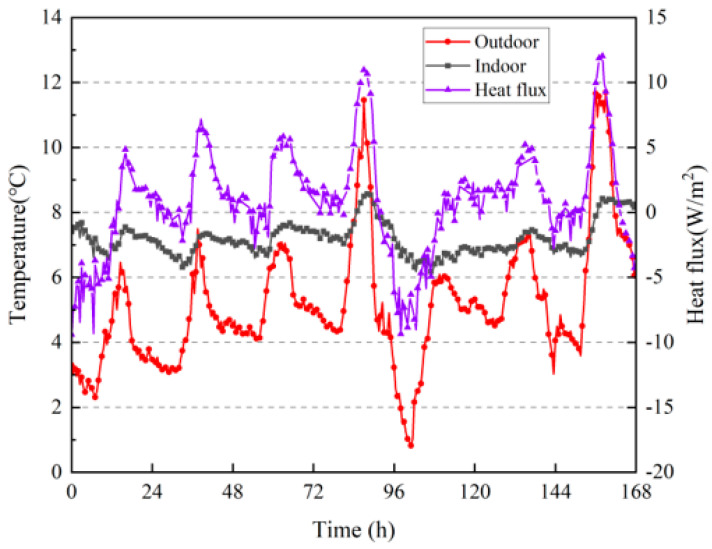
Heat fluxes on the surface inside the wall; indoor and outdoor temperatures.

The heat flux calculation formula for the inner surface of the wall in winter was the same as that for summer, the calculation results are shown in Figure 18. According to Equation (9), the total heat absorption of the inner surface of the wall during the measured period was 667.87 W/m^2^ and the total heat release was 361.96 W/m^2^.

#### 5.1.2. Wall Moisture Transfer

**Figure 19:**
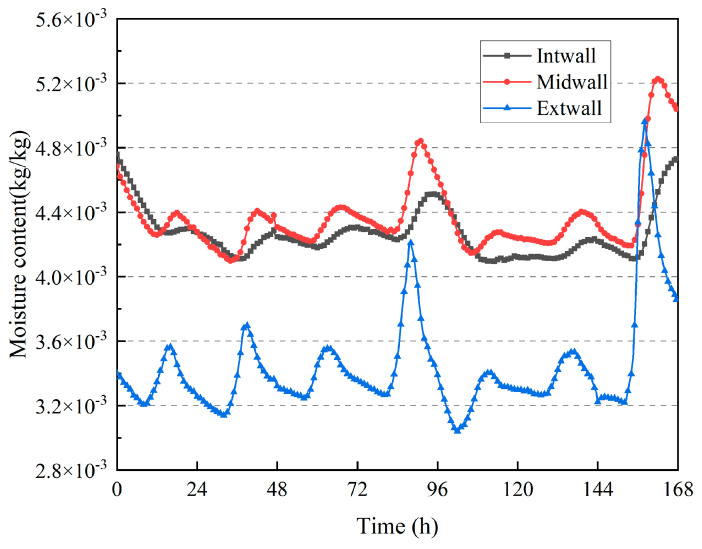
Variation in moisture content inside the wall.

Figure 19 shows the change in moisture content inside the wall, and the fluctuation in moisture content was the same as the fluctuation in temperature at the corresponding position on the wall. The greater the fluctuation in temperature, the greater the fluctuation in moisture content, and the lower the moisture content on the outer surface of the wall. Because of the high outdoor air flow rate, the evaporation of moisture components on the outer surface of the wall was faster, and the outer surface was in the air–drying stage. The highest moisture content on the inner surface was due to the same reason as that in the summer test results, that is, the new wall had a high moisture content. However, compared with the summer test period, the difference in moisture content between the measured points inside the wall was significantly reduced during the winter test period after six months, and the overall moisture content of the wall was much lower than that in summer. This indicated that the rammed–earth wall transferred a large amount of moisture to the outside during the half–year period from summer to winter.

**Figure 20:**
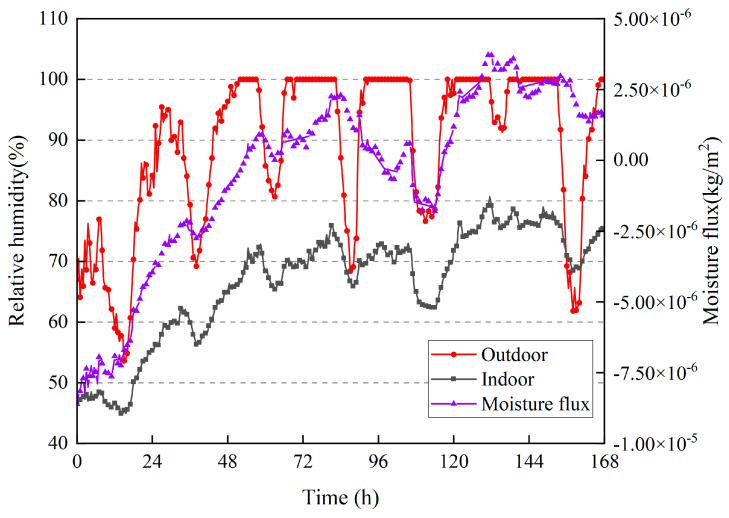
Moisture flux on the surface inside the wall; indoor and outdoor relative humidities.

According to Figure 20, the fluctuation in moisture flux is consistent with the change in indoor humidity, indicating that when the indoor humidity is high, the walls can absorb moisture from the indoor air. When the indoor humidity decreases, rammed–earth walls can disperse moisture into the room, indicating that rammed–earth walls can effectively regulate changes in indoor humidity and maintain a stable indoor humidity environment with good wet–buffering characteristics. Similar to the calculation of the moisture flux in summer, according to Equation (10), the moisture absorption of the wall in winter was 3.3 × 10^−4^ kg/m^2^, and the moisture dissipation was 4.78 × 10^−4^ kg/m^2^.

### 5.2. Indoor Temperature and Humidity Changes

#### 5.2.1. Indoor Temperature

**Figure 21:**
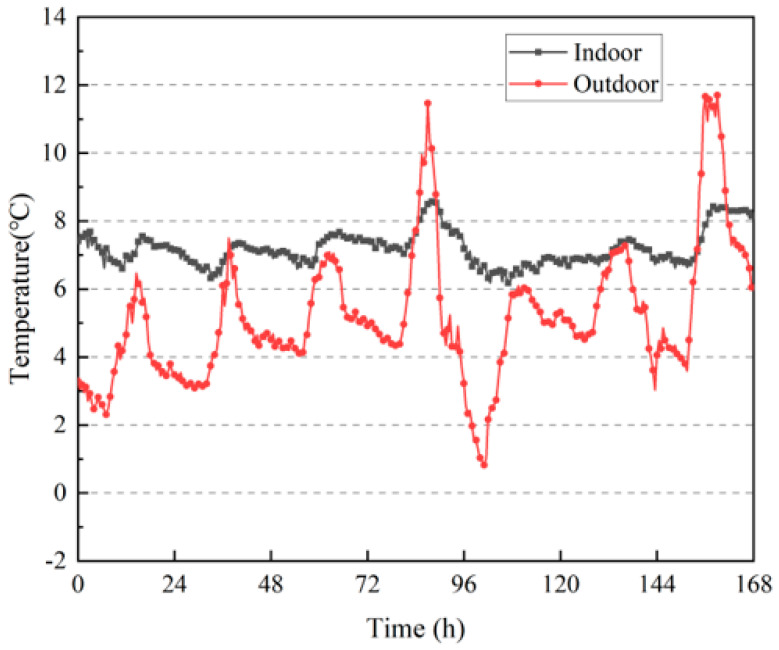
Changes in indoor and outdoor temperatures.

As shown in Figure 21, the outdoor temperature fluctuated widely and was lower than the indoor temperature most of the time, whereas the indoor temperature fluctuation of the rammed–earth building was more stable, and the indoor daily temperature fluctuation still did not exceed 2 °C when the outdoor daily temperature fluctuated up to 8 °C. The highest outdoor temperature was close to 12 °C, and the night temperature was as low as 1 °C, whereas the indoor temperature was always maintained between 6 °C and 9 °C, which indicated that the rammed–earth building also had good heat insulation and thermal stability in winter.

#### 5.2.2. Indoor Humidity

**Figure 22:**
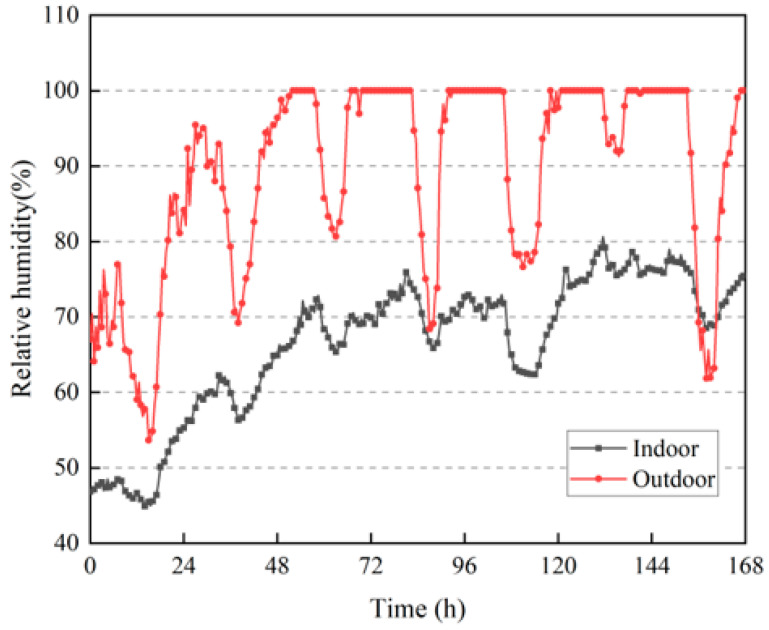
Changes in indoor and outdoor relative humidities.

**Figure 23:**
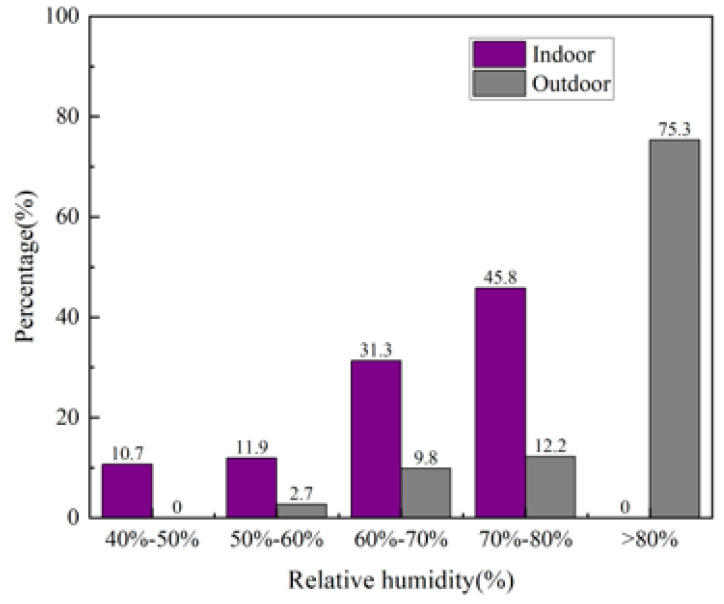
Percentage of indoor and outdoor relative humidity periods.

Figure 22 shows the changes in the indoor relative humidity of the building during the test period. It can be observed from the figure that the outdoor relative humidity fluctuated more, and most of the values were much higher than the indoor values. The overall indoor and outdoor relative humidity was also greater than that in summer; the outdoor relative humidity can reach up to 100%, and the indoor relative humidity can exceed 80% in winter. From the proportion of indoor and outdoor relative humidity periods in Figure 23, it can be observed that the indoor relative humidity was mostly between 60% and 80%, and the proportion of outdoor relative humidity periods greater than 80% was as high as 75.3%. This shows that the rammed–earth building has good humidity–buffering capacity, but the higher indoor and outdoor air relative humidity degrades the durability of the wall and indoor air quality, and can easily cause mold growth.

## 6. Conclusions

In this study, the thermal and physical properties of rammed–earth materials in the northwest region of Sichuan were investigated. The changes in their thermal physical properties under different moisture content conditions, as well as the corresponding functional relationships and isothermal moisture absorption and release curves, were obtained. During summer and winter, the coupled heat and moisture transfer processes of rammed-earth walls and changes in the indoor environment of buildings were analyzed. The main findings of this study are as follows:

(1) The thermal conductivity and other thermophysical parameters of rammed earth under different moisture content conditions were measured, and the equations of the thermal physical parameters as a function of moisture content were fitted, which proved that the higher the moisture content of the rammed–earth materials, the larger the related thermal physical parameters.

(2) The moisture absorption and discharge amounts of the rammed–earth materials were measured under different relative humidities, and the corresponding isothermal equilibrium moisture contents were calculated. The isothermal moisture absorption and discharge curves of the rammed–earth materials were fitted by nonlinear curves, and the results showed that they had good moisture absorption and discharge characteristics, and that the moisture absorption performance was better than the moisture discharge performance.

(3) Under the extremely high–temperature weather conditions during summer, the rammed–earth wall interior surface and building interior environment had lower temperatures and relatively stable temperature and humidity fluctuations. The daily average indoor temperature fluctuation was 3.62 °C, and the daily average relative humidity fluctuation was 12.85%. The new wall continuously dissipated moisture in summer, and the interior relative humidity was in the comfort zone of 50–60% for 72.6% of the time. The fluctuations were more stable, which proves that the rammed–earth building has excellent thermal insulation and buffer characteristics under extremely high-temperature weather and can create a more comfortable indoor environment.

(4) Under the low-temperature and high–humidity conditions during winter, the rammed–earth wall interior surface and building interior environment had more stable temperature and humidity fluctuations, with higher temperatures and lower humidity. The daily average indoor temperature fluctuation was 1.21 °C, and the daily average relative humidity fluctuation was 9.69%. With a change in the indoor relative humidity, the wall interior surface could absorb and release moisture accordingly, effectively regulating the indoor humidity environment. This indicates that the rammed-earth building has excellent thermal insulation performance and humidity–buffering characteristics during winter.

## Figures and Tables

**Table 1:** The relative humidity corresponds to each saturated salt solution.

Saturated Salt Solution	LiCl	MgCl_2_	K_2_CO_3_	NaBr	NaCl	KCl	K_2_SO_4_
*φ* (%)	11.31	32.78	43	57.8	75.3	84.3	97.3

**Table 2:** The average daily temperature inside the wall (°C).

Days	1	2	3	4	5	6	7
Ext-wall	33.79	34.15	34.47	34.58	34.77	35.19	34.75
Mid-wall	32.61	32.82	33.23	33.36	33.53	33.92	33.80
Int-wall	32.08	32.25	32.67	32.83	33.03	33.40	33.35

**Table 3:** Indoor daily maximum temperature lag time.

Days	1	2	3	4	5	6	7
Time (h)	5	8	6	4.5	3.5	5.5	3

**Table 4:** The average daily temperature inside the wall. (°C).

Days	1	2	3	4	5	6	7
Ext-wall	6.13	6.08	6.40	7.15	5.86	6.31	8.14
Mid-wall	6.93	6.39	6.66	7.14	6.55	6.51	7.46
Int-wall	7.42	6.75	6.98	7.34	6.97	6.8	7.34

## Data Availability

Data is contained within the article.

## Nomenclature

*u*	equilibrium moisture content (kg/kg)
*m_i_*	mass of the specimen in equilibrium (kg)
*m*_0_	mass of the specimen when it is completely dry (kg)
*D*	thermal diffusion coefficient (m^2^/s)
*w*	Moisture content by weight (kg/kg)
*C_p,m_*	mass specific heat capacity (J/kg·k)
*q_test_*	heat flux (W/m^2^)
*h_i_*	convective heat transfer coefficient of internal surface (W/m^2^·K)
*T_i_*	indoor temperature (K)
*T_surfi_*	the temperature on the internal surface (K)
*g_test_*	moisture flux (kg/m^2^)
*P_sat,i_*	the indoor partial pressure of saturated vapor (Pa)
*P_sat,surfi_*	the partial pressure of saturated vapor on the internal surface (Pa)
Greek symbols	
*λ*	thermal conductivity (W/m·K)
*ρ_m_*	the density of building materials (kg/m^3^)
*φ*	relative humidity (%)
*φ_i_*	indoor relative humidity (%)
*β_i_*	moisture exchange transfer coefficient of internal surface (s/m)
*φ_surfi_*	relative humidity of internal surface (%)
